# Trust in older persons: A quantitative analysis of alignment in triads of older persons, informal carers and home care nurses

**DOI:** 10.1111/hsc.12820

**Published:** 2019-07-26

**Authors:** Kirti D. Doekhie, Mathilde M. H. Strating, Martina Buljac‐Samardzic, Jaap Paauwe

**Affiliations:** ^1^ Erasmus School of Health Policy & Management (ESHPM) Erasmus University Rotterdam Rotterdam The Netherlands; ^2^ Department of Applied Economics Erasmus University Rotterdam Rotterdam The Netherlands; ^3^ Department of Human Resource Studies Tilburg University Tilburg The Netherlands

**Keywords:** informal carers, primary care, self‐management, triads, trust

## Abstract

Self‐management by older persons could be influenced by the level of trust found in triads of informal carers, formal care providers and care recipient, the older person. Little research has been done on care providers’ trust in older persons. This study aims to explore the level of trust that informal carers and home care nurses have in older persons, the extent of alignment in triads and the relationship between trust in older persons and self‐management. We conducted a cross‐sectional survey study in the Netherlands, sampling 133 older persons, 64 informal carers and 72 nurses, which resulted in 39 triads. Alignment level was analysed through Intraclass Correlation Coefficient 1 scores and absolute and mean difference scores. Correlation analysis and one‐way analysis of variance measured the relationship between trust and self‐management. The results show that triads contain both alignment and misalignment. Misalignment occurs mostly when informal carers and nurses have little trust in the older person while this person views their own behaviour towards their caregivers positively. Care providers’ trust levels relate significantly to their perception of the person's ability to self‐manage, but not to the person's self‐rated ability. This could be explained by care providers not communicating their intrinsic trust in the older person to them. Trust building could be enhanced by organising discussions of mutual expectations of trust and both formal and informal care providers could benefit from compassionate assessment training, to learn how to openly express their trust in the older person.

**What is known about this topic**

- There is a growing emphasis on self‐management for older persons, shaped in triads of older persons, informal carers and formal care providers.
- Trust (the expectation that one can rely on another person) can positively influence an older person's self‐management.
- Research on care providers’ trust in older persons is limited.

**What this paper adds**

- Perceptions of trust both align and misalign in triads of older persons, informal and formal care providers.
- Misalignments arise when care providers have low levels of trust in older persons while those persons view their behaviour towards them positively.
- The level of trust care providers have in the older person relates significantly to their perception of that person's self‐management ability.

## 1 INTRODUCTION

Today, older persons are increasingly expected to manage their own health care and life (Hengelaar et al., 2016). This emphasis on self‐management fits the policy trend in many western European countries to reduce institutionalised secondary care and encourage older persons to live at home for as long as possible in order to contain excessively growing healthcare costs (Broese van Groenou, Jacobs, Zwart‐Olde, & Deeg, 2016; Dahlberg, Demack, & Bambra, 2007; Kutzleben, Reuther, Dortmann, & Holle, 2016; Wittenberg, Kwekkeboom, Staaks, Verhoeff, & Boer, 2017). Self‐management can be defined variously (Barlow, Wright, Sheasby, Turner, & Hainsworth, 2002; van Hooft, Dwarswaard, Jedeloo, Bal, & van Staa, 2015). This study defines self‐management as ‘the individual's ability to manage symptoms, treatment, physical and psychosocial consequences and lifestyle changes inherent to living with a (chronic) condition and to affect the cognitive, behavioural and emotional responses necessary to maintain a satisfactory quality of life’ (Barlow et al., 2002)*.* Put differently, self‐management refers to a patient's attitudes, behaviours and skills to cope with the impact of their (chronic) condition on their daily life, for example by exercising more and making dietary changes, or symptom management such as self‐monitoring glucose levels (Barlow et al., 2002; Lawn & Schoo, 2010).

Older persons do not manage their health in isolation from their social environment. Rather, successful self‐management depends on a person's collaborative relationships with both informal and formal care providers (Dwarswaard, Bakker, Staa, & Boeije, 2016; van Hooft et al., 2015; Whitehead, Jacob, Towell, Abu‐qamar, & Cole‐Heath, 2018). A growing stream of literature focuses on triads of care recipient (older person), informal and formal care providers and how the quality of their relationships influences self‐management (Adams & Gardiner, 2005; Bovenkamp & Dwarswaard, 2017; Hengelaar et al., 2016; Lindahl, Lidén, & Lindblad, 2011; Wiechula et al., 2016). Self‐management is, for example, conceptualised as ‘mutual participation between patients and caregivers’ and ‘the conjunction with family, community and healthcare professionals’ (Richard & Shea, 2011). Lindahl and colleagues (2011) emphasise the importance of building friendships between triad members to ensure that the recipient's needs are met and to develop stable relationships in which all involved treat each other as equals.

Trust is a keystone in triads of older persons, informal carers and formal care providers (Hall, Zheng, et al., 2002; LoCurto & Berg, 2016; Pelaccia et al., 2016; Thorne & Robinson, 1988; Wiechula et al., 2016). It is closely related to other important aspects of relationships such as satisfaction, communication and privacy (Hall, Dugan, Zheng, & Mishra, 2001). Older persons’ trust in their care providers can influence their involvement in decision‐making (Brown et al., 2002; Kraetschmer, Sharpe, Urowitz, & Deber, 2004) and perceived self‐management ability (Bonds et al., 2004; Gabay, 2015; Young, Len‐Rios, Brown, Moreno, & Cox, 2017). Research shows that trust between older persons and their care providers is important for continuity of care, patient satisfaction and adherence to therapeutic recommendations (Brennan et al., 2013; Calnan & Rowe, 2008; Hall, Camacho, Dugan, & Balkrishnan, 2002; Kramer & Cook, 2004; LoCurto & Berg, 2016). Most studies define trust as ‘an expectation that the other person will behave in a way that is beneficial, or at least not harmful, and allows for risks to be taken based on this expectation’ (Brennan et al., 2013; LoCurto & Berg, 2016; Mascarenhas et al., 2006; Moskowitz et al., 2011; Thom et al., 2011). In other words, to trust a person means expecting that their behaviour and word, promise or statement can be relied upon (Mascarenhas et al., 2006).

However, most studies look solely at the trust that the care recipient has in their care providers (Brennan et al., 2013; Thom et al., 2011; Wilk & Platt, 2016). Research on trust in the older person‐care provider relationships from both angles is scarce with most studies mentioning only briefly the care providers’ trust in (older) persons (Brennan et al., 2013; Kramer & Cook, 2004). A review by Wilk and Platt (2016) identified just two empirical articles on trust in older persons from the care providers’ perspective (Moskowitz et al., 2011; Thom et al., 2011).

As the concept of trust is embedded in the social context of triad interaction, the perspectives of care providers should be embraced (Brennan et al., 2013; Pelaccia et al., 2016; Thom et al., 2011; Thorne & Robinson, 1988; Wilk & Platt, 2016). The trend in older person self‐management has redefined a paternalistic older person–care provider relationship with a patient‐driven and patient‐centred one (Brennan et al., 2013; Douglass & Calnan, 2016; Murray & McCrone, 2015; Pelaccia et al., 2016; Wilk & Platt, 2016). Thus care providers need to trust that the older person will behave in ways that benefit their own health, such as following a treatment plan or giving accurate information about their condition (Pelaccia et al., 2016; Thom et al., 2011; Thorne & Robinson, 1988). Trust should not be blindly assumed. Rather, the older person has to show that they can ‘earn’ their care providers’ trust (Brennan et al., 2013). Meanwhile, research suggests that care providers’ trust could influence the behaviour of older persons (Calnan & Rowe, 2008; Kim, Kaplowitz, & Johnston, 2004; Thom et al., 2011). Rogers (2002) argues that by expressing trust, care providers positively contribute to the older person's confidence in managing their health. One could argue that it is desirable to align the care provider's trust and the older person's view of their own trustworthiness. Research by Pelaccia and colleagues (2016) shows that some formal care providers label certain older persons as ‘unreliable’ and trust them less because, for example, they do not think they are being sincere or honest in answering questions, whereas the older persons feel that they are.

### 1.1 Aims of this study

This study is one of the first to shed the light on trust in older persons from three perspectives: informal carers, home care nurses and older persons themselves*.* We explore the interpersonal character of trust in triads from multiple perspectives, showing how trust exists and occurs in relationships between specific persons, rather than focussing on merely a single perspective (Kenny, Kashy, Cook, & Simpson, 2006). We aim to gain deeper insight into the concept of trust in older persons within the triad, and how trust relates to self‐management ability. In doing so, we explore trust and its relationship with self‐management from all three individual perspectives, and also on the level of alignment in trust between the three triad groups and how (mis)alignment relates to self‐management.

## 2 METHODS

### 2.1 Design and participants

We conducted a cross‐sectional survey study in the Netherlands. The questionnaire focused on *interpersonal* trust between the care recipient and their informal carers and nurses and excludes institutional or systemic trust (in collective entities or organisations) (Douglass & Calnan, 2016; Hall, Zheng, et al., 2002). The study population consisted of triads of older persons, their informal carers and the most‐involved home care nurse. Nurses were selected as the formal care provider group, as older persons living at home often receive home care and thus interact often with these nurses (Lindahl et al., 2011). Trusting relationships are important in these care triads (Lindahl et al., 2011; Weman & Fagerberg, 2006; Wiechula et al., 2016).

All older persons received care from one large home care organisation active in many cities in the west and south of the Netherlands. Inclusion criteria were: aged 60 or older and receiving home care at least 2 days per week for 6 months or longer. All nurses worked for the same home care organisation. Informal carers were defined as family members (partners or children), close friends or neighbours who provide non‐professional, voluntary care (Janse, Huijsman, Looman, & Fabbricotti, 2018).

Older persons gave written informed consent to participate in the study. For the informal carers and nurses, informed consent was assumed by their completion of the questionnaire. All questionnaires were filled in anonymously. The three participants belonging to a triad were given a unique identification number to distinguish triads. The names linked to these numbers were saved in an encrypted file that was made available to the main researcher (KD) only.

### 2.2 Data collection

Data were collected between July 2016 and December 2017. The home care organisation randomly selected 2000 older persons in their database, based on two criteria: (a) persons aged 60 years or older and (b) receiving care at least 2 days per week (Figure 2). An informative letter and consent form were sent to the selected persons. The home care organisation forward contact details to the research team only when the older person had given informed written consent (*n* = 343). At no point did the researchers have access to contact details without the older persons’ written consent. Reasons for not giving consent and any background information on non‐responders could not be collected to ensure their privacy.

KD and two other researchers visited the older persons at home, collecting data through structured interviews, audiotaped with informed consent. The interviews were recorded as most participants gave elaborated on their personal situation. The tapes were used only in the data analysis phase to gain a better understanding of the given scores.

The researchers asked if and from whom the older person received informal care (*n* = 89) and handed over a questionnaire for the informal caregiver, including an informative letter and return envelope. The questionnaires for nurses were distributed through their team leaders. The nurse's informative letter stated the name of the older person concerned in the questionnaire and a return envelope. Seventeen nurses received separate questionnaires for two older persons; one nurse received questionnaires for three persons.

### 2.3 Instruments

**Figure 1:**
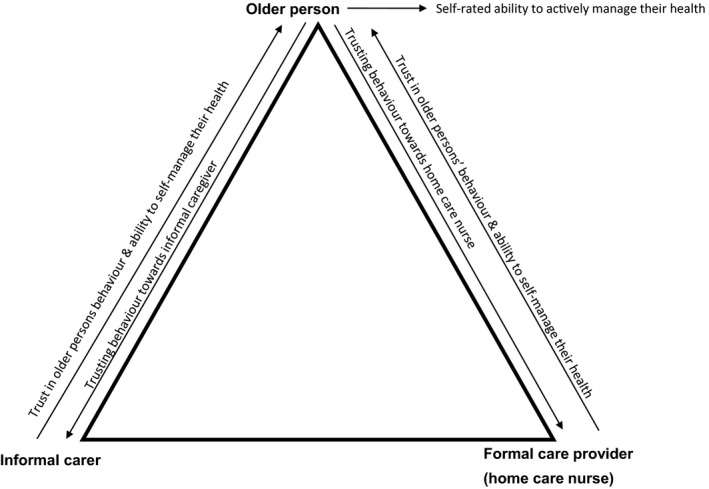
Trust and self‐management in the questionnaires. Illustration of how the concepts of trust and self‐management were measured in the questionnaires

Different questions were designed for each respondent group, focussing on two aspects: (a) care providers’ trust in older persons and the older person's view of their own behaviour and (b) the self‐management ability of the older person (Figure 1). Examples of descriptive variables in the older persons’ questionnaire are as follows: age, gender, marital status, educational status and health status. Health status was assessed by the validated five‐dimensional, three level EuroQol instrument (EQ‐5D‐3L) and the EuroQol visual analogue scale (EQ VAS) (Szende, Janssen, & Cabases, 2014). The EQ‐5D‐3L dimensions are: mobility, self‐care, usual activities, pain/discomfort and anxiety (Szende et al., 2014). The EQ VAS was used to rate older persons’ self‐rated health on a vertical analogue scale ranging from 0 (worst imaginable health state) to 100 (best imaginable health state) (Szende et al., 2014).

Descriptive variables in the informal carers’ questionnaire were: age, gender, marital status, educational status, living status, relationship with person and type of care/support for the person. Descriptive variables in the nurse questionnaire were as follows: age, gender, educational status, number of years nursing experience and number of years involved in caring for this older person.

The 12‐item Physician Trust in the Person scale (Thom et al., 2011) measured the trust level in older persons of the informal carer and nurse as well as the person's assessment of their own behaviour. The first to measure trust *in* a patient by a formal care provider (Brennan et al., 2013; Thom et al., 2011), this scale is designed to gain insight into an under‐researched topic (i.e. trust in patients) and a better understanding of how trust in patients and the processes in a care provider–patient relationship could lead to improvements in quality of care and care provider and patient satisfaction. Each item reflects on a specific type of behaviour of the older person towards the care provider, in terms of the patient's role in the relationship (e.g. whether patients understand what they are told) and the patient's respect for personal boundaries (e.g. whether patients make unreasonable demands) (Thom et al., 2011).

Three items with regard to informal care were excluded from the informal carers’ and the older persons’ questionnaires: provide information on used medicines, manipulate the office visit for secondary gain, and keeps their appointments. The first item was excluded based on insights from the research team's prior studies on the older person–informal carer relationship, which indicate that patients rarely discuss their medicine list with their informal carer. The other two items were excluded as informal carers provide care at home, not in an office, and care delivery is often ad‐hoc and not primarily arranged to suit the informal carer's schedule. The latter two were also excluded from the home care nurses’ questionnaire, as home care is provided at a person's home and not in an office and within specific time frames and not by scheduled appointments that patients have to keep like in hospitals.

The informal carer and nurse were asked how confident they were in the person's behaviour towards them, using a 5‐point scale ranging from 1 (not at all confident) to 5 (completely confident). An item in the informal carer questionnaire was: ‘How confident are you that this person will tell you about a major change in their condition?’ An item in the nurse questionnaire was: ‘How confident are you that this person will respect your personal boundaries?’ Cronbach's alpha for the informal carer‐adapted scale was .91 and for the nurse‐adapted scale was .91.

For the older person questionnaire, the Physician Trust in Patient scale items were reformulated to statements, so that patients would assess behaviour towards either the informal carer or nurse [hereafter referred to as: patient's behaviour]. Each question was duplicated so that older persons could rate their answers for both informal carer and nurse on a 5‐point scale ranging from 1 (completely disagree) to 5 (completely agree). Questions included ‘I tell my informal carer if there are major changes in my condition’ and ‘I respect the personal boundaries of the home care nurse’. Cronbach's alpha for the older person‐adapted scale was .71 for informal caregivers and .64 for the nurse.

Self‐management was measured using one of the nine scales of the Health Literacy Questionnaire instrument (HLQ): the ‘ability to actively manage my health’ scale (Osborne, Batterham, Elsworth, Hawkins, & Buchbinder, 2013). The HLQ addresses health literacy as a multidimensional concept and contains nine validated scales that measure distinct dimensions of health literacy and can be used independently to measure a distinct dimension of health literacy (Osborne et al., 2013). The scale this study used focuses on the extent to which persons take responsibility for their own health (Osborne et al., 2013).

This study used the original ‘ability to actively manage my health’ scale for the older persons, whereas it rephrased items for the care providers, using a 4‐point scale ranging from 1 (‘strongly disagree’) to 4 (‘strongly agree’) on all three questionnaires. One item in the older person questionnaire was: ‘I plan what I need to do to be healthy’ and in the care providers' questionnaires: ‘This person makes time to be healthy despite having other things in their life’. Cronbach's alphas were .87 for the older person questionnaire, .92 for the informal carer questionnaire and .94 for the nurse questionnaire.

### 2.4 Data analysis

Data were analysed using IBM SPSS 23.0. Regarding the first aim, the level of alignment between perceptions of trust was calculated in two ways. Firstly, we calculated the Intraclass Correlation Coefficient 1 (ICC1) scores to determine the level of agreement between the care providers’ levels of trust in the person in relation to the person's view of their own trusting behaviour. The strength of agreement reflected by ICC1 was labelled in concordance with other research: ≤0.40 poor to fair agreement, 0.41–0.60 moderate agreement, 0.61–0.80 good agreement, and 0.81–1.00 excellent agreement (Landis & Koch, 1977; Poort et al., 2016). Secondly, for each triad, we calculated difference scores of trust using the absolute scale scores on the Physician Trust in the Patient scale. Sum scores per respondent group were first calculated. For example, in the patient questionnaire, their behaviour towards the informal carer and the latter's level of trust were measured with nine items of the scale, thus the sum score was the total of all nine items. Next, the sum scores of two respondent groups were subtracted to calculate the difference scores. To calculate the empirical range of difference scores, the minimum and maximum sum scores per respondent group were first calculated. Given the use of a 5‐point scale, the minimum sum score for the older persons’ view of their own behaviour towards the informal carer and the latter's level of trust sum score is nine (scoring of one on all nine items) and their maximum sum score is 45 (scoring of five on all nine items). Therefore, the minimum difference score is the situation in which the informal carer would have a minimum score and the older person would have the maximum score, leading to a score of −36 (9 minus 45) and the maximum score is the reversed situation, leading to a score of +36 (45 minus 9). The difference scores and empirical ranges were calculated in a similar manner for the other relationships in the triads.

Pearson correlation analysis was used to assess the relationship between the difference scores. Next, each triad was categorised as an alignment or misalignment based on the mean difference score for each dyadic relationship in the triad (informal carer–older person; nurse–older person; nurse–informal carer).

One‐way analysis of variance (ANOVA) was used to analyse the significant difference per dyadic relationship between the alignment categories in their mean difference trust scores.

Regarding the second aim, we used correlation analysis to analyse the relationship between both care providers’ mean levels of trust and persons’ self‐management ability. One‐way ANOVA analysis was used to analyse the relationship between the difference scores and person's self‐management ability.

## 3 RESULTS

### 3.1 Sample characteristics quantitative results

**Figure 2:**
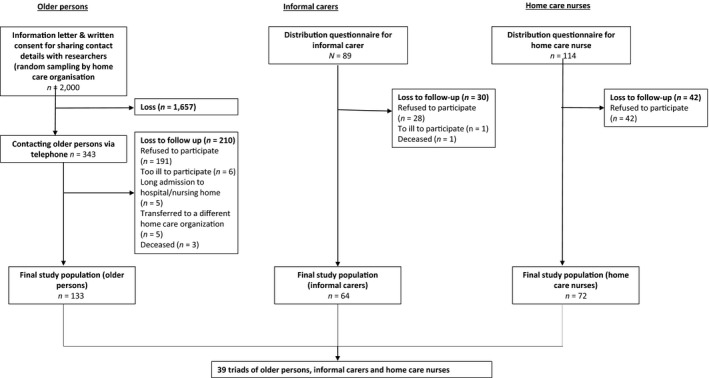
Flow chart data collection study. Description of the data collection process and the number of participants per stage

In total, 133 older persons, 64 informal carers and 72 home care nurses filled in the questionnaire. Based on these responses, we identified and analyzed 39 triads (Figure 2).

Table 1 provides the descriptives of the sample. The average age of the older persons was 80.4 years, 41% was male and the majority was Dutch (94.9%). Around half of the older persons were widows or widowers (53.8%), lived alone (59%) and 16 respondents (41.03%) had a co‐resident informal carer. Most persons suffered from Parkinson's disease (69.20%) and/or had a visual disability (51.30%). The percentage of male older persons and persons living alone is comparable with the Dutch older population (Statistics Netherlands [CBS], 2017, 2018). However, the sample did not represent the ethnic diversity of the Dutch older population, for example the large group of Turkish and Surinamese persons (CBS, 2018). The EQ‐5D‐3L utility score and the EQ5D VAS score were 0.50 and 55.64 respectively. One sample test showed that both scores are significantly lower than Dutch population norms (*t* = −7.77, *p* < .05 and *t* = −13.28, *p* < .05 respectively) (Szende et al., 2014).

**Table 1:** Descriptives of triads (*n* = 39)

	Persons		Informal carers		Home care nurses	
	*N*	%	*N*	%	*N*	%
Age, mean (*SD*)	80.39 (7.98)		62.61 (15.54)		44.60 (11.43)	
Sex						
Male	16	41.00	12	31.60	1	2.60
Marital status						
Married	13	33.30	25	65.80		
Unmarried	0	0	6	15.80		
Divorced	3	7.70	2	5.30		
Widow/widower	21	53.80	3	7.70		
Registered partnership	2	5.10	2	5.30		
Educational status						
Less than secondary school	8	20.50	9	23.70	0	0
Secondary school/technical school	29	74.40	24	63.20	35	92.10
College or above	2	5.10	5	13.20	3	7.90
Living status						
Alone	23	59.00	9	23.70		
With partner	15	38.50	22	57.90		
With partner and children	0	0	5	13.20		
With children	1	2.60	1	2.60		
With parent			1	2.60		
Ethnic background						
Dutch	37	94.90	37	94.90		
British	1	2.60				
Indonesian	1	2.60				
Aruban			1	2.60		
Canadian			1	2.60		
Total number of co‐resident informal carers	16	41.03				
of which partner	15	93.75				
of which son/daughter (in law)	1	6.25				
**EQ‐5D‐3L dimensions**						
Mobility						
No problems	6	15.40				
Some problems	32	82.10				
Extreme problems	1	2.60				
Self‐care						
No problems	19	48.70				
Some problems	12	30.80				
Extreme problems	8	20.50				
Usual activities						
No problems	10	25.60				
Some problems	18	46.20				
Extreme problems	11	28.20				
Pain/discomfort						
No problems	9	23.10				
Some problems	19	48.70				
Extreme problems	11	28.20				
Anxiety/depression						
No problems	27	69.20				
Some problems	10	25.60				
Extreme problems	2	5.1				
EQ‐5D utility scores mean (*SD*)						
EQ‐5D‐3L utility score	0.50 (0.29)					
EQ5D‐VAS	55.64 (13.33)					
Chronic condition in the past 12 months (multiple options possible)						
Diabetes	12	30.80				
Damage due to a stroke	8	20.50				
Heart failure	12	30.80				
Cancer	6	15.40				
Asthma, COPD, bronchitis	17	43.60				
Arthroses	27	69.20				
Osteoporosis	11	28.20				
Parkinson's disease	1	2.60				
Problems with stability	20	51.30				
Hearing disability	17	43.60				
Visual disability	11	28.20				
Depression	8	20.50				
Average days per week home care, mean (*SD*)	4.58 (2.66)					
Average number of nurses per week, mean (*SD*)	5.38 (3.11)					
Average time (in minutes) per nurse visit, mean (*SD*)	19.31 (13.03)					
Relationship with older person						
Partner			14	37.81		
Son/daughter (in law)			18	48.60		
Grandson/granddaughter (in law)			1	2.70		
Nephew/niece/cousin			1	2.70		
Friend			2	5.40		
Neighbour			1	2.70		
Type of informal care (multiple options possible)						
Bathing and getting dressed			4	10.50		
Meal preparation			16	42.10		
Daily care			9	23.70		
Medication provision			8	21.10		
Housework			24	63.20		
Grocery shopping			32	84.20		
Administrative support			18	47.40		
Transport to doctor's office			26	68.40		
Number of years active as home care nurse						
Under 10					19	50
Between 10 and 25					17	44.70
More than 25					2	5.30
Number of years involved in caring for person						
Under one					9	23.70
Between one and three					24	63.20
More than three					5	13.20

Abbreviation: *SD* = standard deviation.

*N* = 38 due to missing data for one informal carer.

*N* = 38 due to missing data for one nurse.

*N* = 37 due to missing data for two home care nurses.

The average age of the informal carers was 62.6 year, 31.6% was male and the large majority was Dutch (94.9%). Informal carers were mostly married (65.8%) and about half were a son or daughter (in law) of the older person (48.6%). Informal carers provided household support (63.2%), grocery shopping (84.2%) and transport to the doctor's office (68.4%). Compared to the Dutch population, the informal carer sample is largely representative as 56% is female and 42% provides care for their parent (in law) (Klerk, Boer, Plaisier, & Schyns, 2016). The range of activities is comparable to the Dutch population, although the percentage of household support and transportation is slightly lower in the Dutch population (45% and 53% respectively) (Klerk et al., 2016).

Only one nurse was male (2.6%) and most nurses had looked after the person for between 1 and 3 years (63.2%). The large group of female nurses is representative for all the nurses in the participating home care organisation.

### 3.2 Trust mean scores

**Figure 3:**
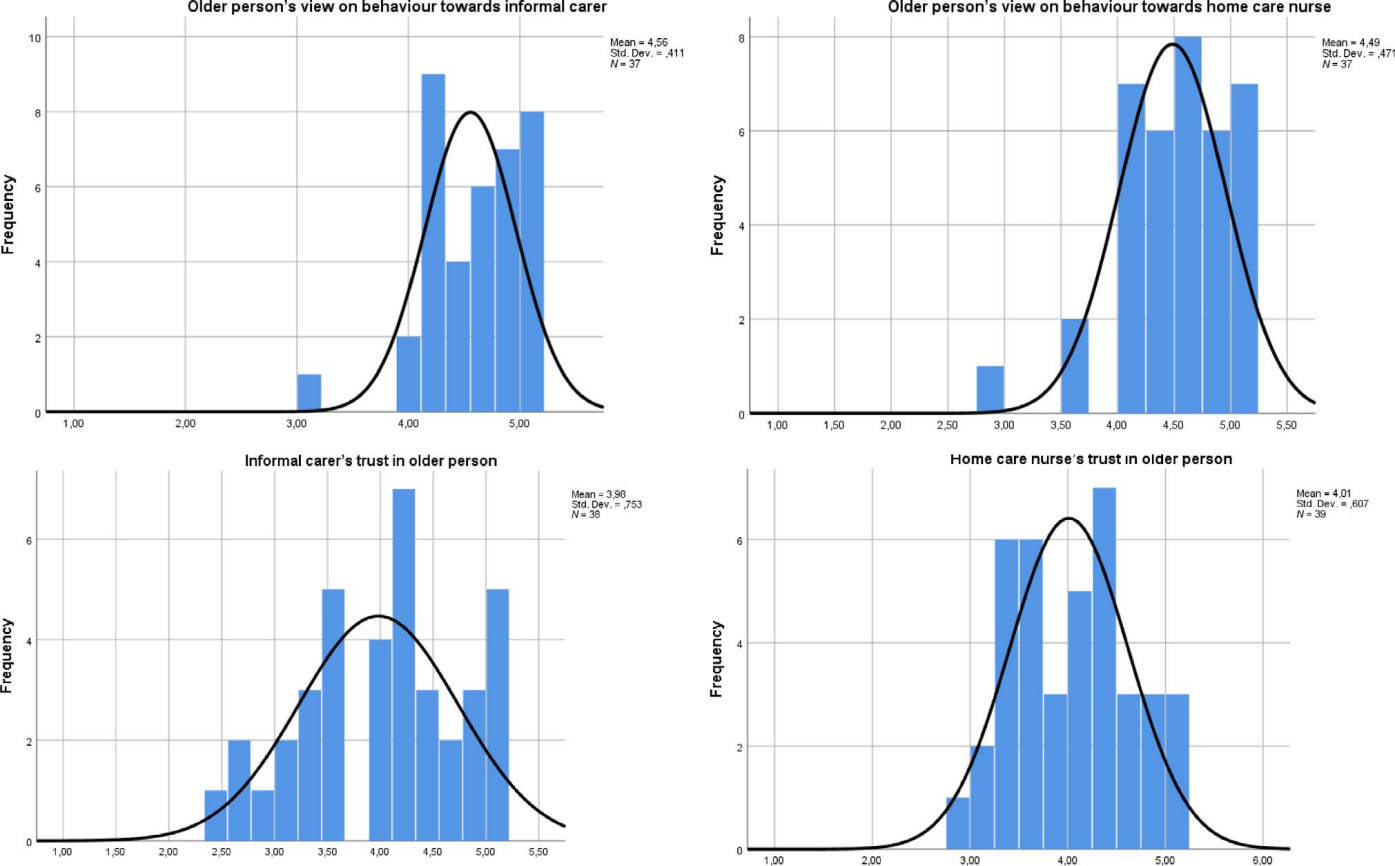
Histogram distribution trust scores from all three respondent groups. Plots with the normal distribution of the trust scores from older persons, informal carers and home care nurses [Colour figure can be viewed at https://www.wileyonlinelibrary.com]

On a scale of 1–5, Kolmogorov–Smirnov Test of Normality showed a normal distribution of the informal carers' and nurses' trust scores as well as the older person's score with regard to the nurse (Figure 3). For the older person's score regarding the informal carer, the significance level was .05, suggesting a relatively normal distribution (Figure 3). The mean informal carer's level of trust was 3.98 (*SD* = 0.75) and the mean nurse's level of trust in the older person was 4.01 (*SD* = 0.61). The older person's mean score on their behaviour, meaning the mean scores on the reformulated Physician Trust in the Patient scale [hereafter referred to as: patients view of their own behaviour] towards the informal carer was 4.56 (*SD* = 0.41) and towards the 4.49 (*SD* = 0.47) (Table 2).

**Table 2:** Means and correlational analysis of trust, difference scores and self‐management

Variable	*N*	Mean	*SD*	1	2	3	4	5	6	7	8	9	10
*Trust variables*													
1. Informal carer's trust in older person	38	3.98	0.75		0.22	0.20	0.16				0.18	0.47*	0.47*
2. Home care nurse's trust in older person	39	4.01	0.61			−0.03	−0.00				−0.99	0.20	0.40**
3. Older person's view on behaviour towards informal carer	37	4.56	0.41				0.49*				0.51*	0.28	0.21
4. Older person's view on behaviour towards home care nurse	37	4.49	0.47								0.37**	0.10	0.13
*Difference scores on trust*													
5. Trust informal carer–older person	38	−4.18	10.19						0.012	−0.50*	−0.00	0.24	0.20
6. Trust home care nurse–older person	39	−1.97	11.62							0.29	−0.20	−0.031	0.12
7. Trust home care nurse–informal carer	38	4.26	8.04								−0.23	−0.26	−0.08
*Outcome variable self‐management*													
8. Older person's self‐rated self‐management ability	39	2.71	0.51									0.40**	0.33**
9. Informal carer's perspective on self‐management	38	2.82	0.58										0.38**
10. Home care nurse's perspective on self‐management	38	3.00	0.44										

Note

Rows 1–4 are the mean difference scores on the trust scale (within the range of 1–5) per respondent group. Rows 5–7 represent the mean difference scores per dyadic relationship (within the empirical ranges), based on the sum scores on the trust scale.

*n* = 38 due to missing data for one informal carer.

*n* = 37 due to missing data for two older persons. In both cases, respondents did not fill in the whole scale.

* Correlation is significant at .01 level (two‐tailed).

** Correlation is significant at .05 level (two‐tailed).

**Figure 4:**
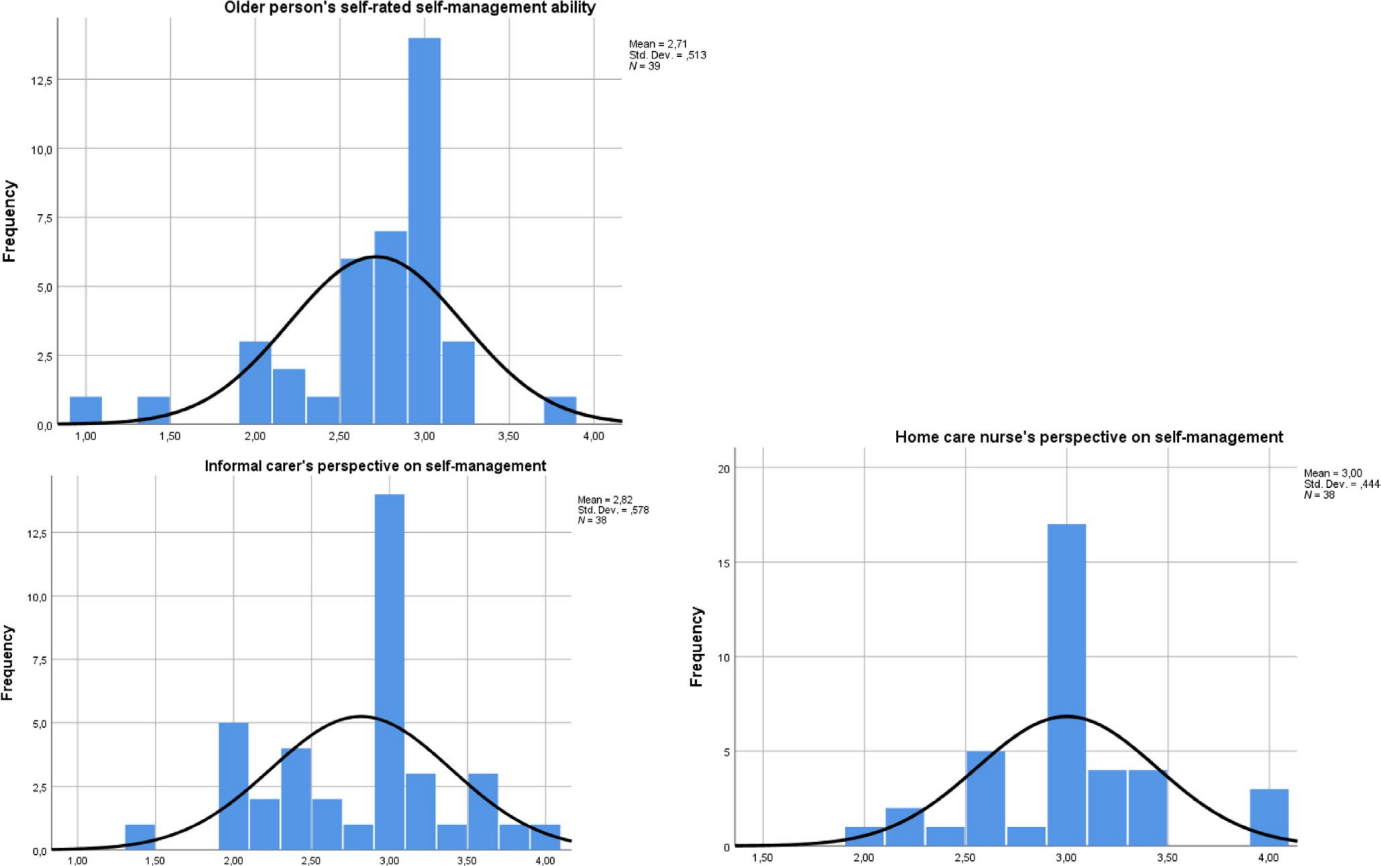
Histogram distribution self‐management scores from all three respondent groups. Plots with the normal distribution of the self‐management scores from older persons, informal carers and home care nurses [Colour figure can be viewed at https://www.wileyonlinelibrary.com]

On a scale of 1–4, the Kolmogorov–Smirnov test showed that self‐management scores were not statistically normally distributed, although the histograms suggest a relatively normal distribution (Figure 4). The older person's self‐rated self‐management mean score was 2.71 (*SD* = 0.51), the informal carer's perspective on the person's self‐management was 2.82 (*SD* = 0.58) and the nurse's perspective was 3.00 (*SD* = 0.44) (Table 2).

### 3.3 Level of alignment between informal carers, nurses and older persons

#### 3.3.1 ICC1—Level of agreement

**Figure 5:**
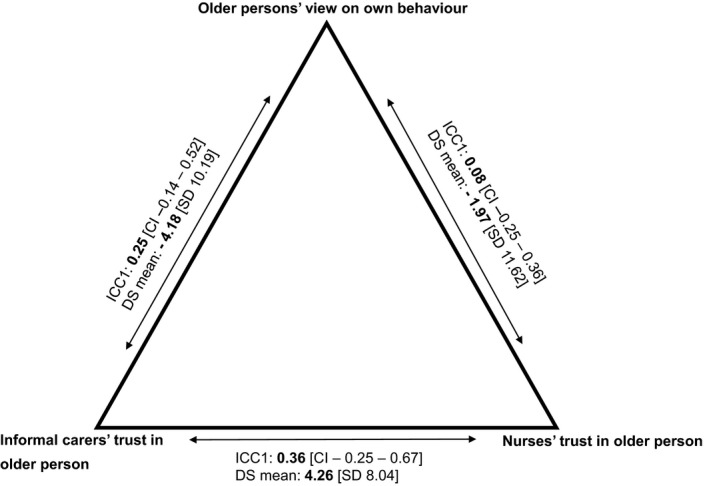
ICC1 and absolute difference scores. Each arrow represents the relationship between two respondent groups (e.g. older persons and nurses). For each relationship, the first number shows the Intraclass Correlation Coefficient 1 score (ICC1) and the corresponding confidence interval (CI). The second number shows the mean difference score (DS) and corresponding standard deviation (*SD*)

The ICC1 level (Figure 5) for the informal carer's trust in the person in relation to the person's view of their own behaviour was 0.25 with a 95% confidence interval from −0.14 to 0.52. This indicates a non‐significant, fairly low level of agreement between informal carers and older persons. The ICC1 score for the nurse's trust in the person in relation to the person's view of their own behaviour was 0.08 with a 95% confidence interval from −0.25 to 0.36. This indicates a non‐significant, very low level of agreement between nurses and older persons. The ICC1 score for the informal carer's trust in relation to the nurse's trust was 0.36 with a confidence interval ranging from −0.25 to 0.67, indicating a non‐significant, fairly low level of agreement.

#### 3.3.2 Difference scores on trust in persons

Difference scores for the level of trust in older persons were calculated for each triad using the absolute value of the difference between the informal carer's and nurse's trust in the person and the person's view of their own behaviour towards both kinds of care provider (Figure 3 and Table 2). Difference scores for the informal carer and older person were calculated with the absolute scores of the informal carer minus the persons’ absolute scores (empirical range between −36 and +36). Difference scores for the nurse and the older person were calculated with the absolute scores of the nurse minus the absolute scores of the person (empirical range between −40 and +40). Difference scores between the nurse and informal carer were calculated with the absolute scores of the nurse minus the informal carer's scores (empirical range between −35 and +41).

Between the informal carer and the older person and between the nurse and the older person, negative mean difference scores were found (Table 2) (−4.18 and −1.97 respectively). This means that the level of trust of the care provider in the person is lower than the person's view of their own behaviour towards both care providers. Between the nurse and informal carer, the mean difference score was 4.26, implying that on average the nurse had more trust in the older person than the informal carer.

Correlation analysis (Table 2) revealed a significant negative correlation between the difference score of the informal carer and older person and the difference score between the nurse and informal carer (*r.* = −.50; *p* < .01). This means that the level of alignment between the informal carer and the older person negatively influences the level of alignment between the nurse and informal carer, and vice versa.

The difference scores for each triad were grouped in three categories to gain more insight into the level of alignment and misalignment in triads (Table 3). Categories were based on the mean difference score of each dyadic relationship in the triad. One‐way ANOVA showed significant differences per dyadic relationship between the three alignment categories in their mean difference score. Between the informal carer and older person, misalignment was found in 18 triads in which the informal carer had a lower level of trust than the person's view of their own behaviour (mean = −11.39, *SD* = 4.67), but another 18 triads showed alignment (mean = −0.06, *SD* = 2.24). Between the nurse and older person, six triads had an alignment and 23 triads showed misalignment with the nurse having a lower level of trust in the person than the person's view of their own behaviour (mean = −8.91, *SD* = 4.80). Between the nurse and informal carer, 14 triads had alignment and 19 triads had misalignment in which the nurse's level of trust in the person was higher than the informal carer's level of trust (mean = 10.37, *SD* = 5.04).

**Table 3:** Level of alignment and misalignment in the triads

	Informal carer–person dyad			Home care nurse–person dyad			Home care nurse–informal carer		
	Ic < p	Alignment	Ic > p	Hcn < p	Alignment	Hcn > p	Hcn < ic	Alignment	Hcn > ic
*N*	18	18	2	23	6	10	5	14	19
Mean difference score (*SD*)	−11.39 (4.67)	−0.06 (2.24)	23.50 (24.79)	−8.91 (4.80)	−0.17 (0.75)	12.90 (11.95)	−9.20 (3.35)	0.79 (2.89)	10.37 (5.04)
Min/max	−23.00/−5.00	−4.00/4.00	6.00/41.00	−20.00/−3.00	−1.00/1.00	4.00/40.00	−13.00/−5.00	−4.00/4.00	5.0/25.00
*F‐*statistic (*p*‐value)	45.47 (0.00*)			33.51 (0.00*)			51.21 (0.00*)		

Note

Categories were based on the average difference scores in each dyadic relationship.

Abbreviations: Ic = Informal carer; p = older person; Hcn = home care nurse.

*N* = 38 for the informal carer–person dyad and home care nurse–informal carer dyad because data are missing for one informal carer.

* *F‐*statistic significant at .01 level.

The descriptive characteristics of the triads per (mis)alignment category are provided in Table S1. In the relationship between informal carers and older persons, the misalignment category in which informal carers' had a higher level of trust than the older person's view of their own behaviour, none of the informal carers was a partner or co‐resident. In the relationship between the nurse and older person, the alignment category is characterised by patients with higher EQ‐5D‐3L scores (0.69) and a lower average time per home care visit (9.67 min) than the two misalignment categories (EQ‐5D‐3L scores 0.48 and 0.45; average time per home care visit (20.44 and 22.50 min respectively). In the relationship between nurses and informal carers, the patient's EQ‐5D‐3L score was substantially lower in the alignment category (0.38) than in the other two categories (0.59 and 0.60 respectively).

### 3.4 Self‐management ability of the person

Correlation analysis was performed to analyse the relationship between the trust variables and self‐management (Table 2). Significant relationships were found between the older person's view of their own behaviour towards the informal carer and the nurse and the person's self‐rated self‐management ability (*r*. = .51, *p* < .01; *r.* = .37, *p* < .05 respectively). This means that when older persons had a positive view of their own behaviour towards either care provider they also had more confidence in their self‐management ability and vice versa.

Significant relationships were found between the informal carer's level of trust and their perception of the person's self‐management ability (*r*. = .47, *p* < .01) and with the perception of the nurse of the person's self‐management ability (*r*. = .47, *p* < .01). For the nurse's level of trust, a significant correlation was found with their perception of the person's self‐management ability (*r.* = .40, *p* < .05).

#### 3.4.1 Difference scores and self‐management of the person

No significant relationships were found between the absolute difference scores and the person's self‐management ability in all three perspectives (Table 2). Regarding the alignment categories, one‐way ANOVA (Table 4) showed no significance in the mean self‐management scores of all three perspectives, with the exception of a significantly higher self‐management score in the informal carer's perspective in alignment between the nurse and informal carer (*F* = 5.70, *p* < .01).

**Table 4:** One‐way ANOVA of self‐management and alignment categories per dyadic relationship

Outcome variable: self‐management	Informal carer–person dyad			Home care nurse–person dyad			Home care nurse–informal carer dyad		
	Ic < p	Alignment	Ic > p	Hcn < p	Alignment	Hcn > p	Hcn < ic	Alignment	Hcn > ic
Older person's self‐ rating									
*N*	18	18	2	23	6	10	5	14	19
Mean (*SD*)	2.78 (0.38)	2.63 (0.66)	2.80 (0.28)	2.83 (0.32)	2.63 (0.37)	2.48 (0.83)	2.88 (0.23)	2.76 (0.38)	2.63 (0.65)
*F‐*statistic	0.37			1.83			0.53		
*p*‐value	0.70			0.18			0.59		
Informal carer's perspective									
*N*	18	18	2	22	6	10	5	14	19
Mean (*SD*)	2.66 (0.49)	2.96 (0.66)	3.00 (0.00)	2.79 (0.47)	3.00 (0.74)	2.76 (0.72)	2.80 (0.35)	3.17 (0.38)	2.56 (0.62)
*F‐*statistic	1.35			0.36			5.70		
*p*‐value	0.27			0.70			0.01*		
Home care nurse's perspective									
*N*	17	18	2	22	6	10	5	14	18
Mean (*SD*)	2.88 (0.37)	3.13 (0.50)	2.70 (0.14)	2.96 (0.41)	3.10 (0.47)	3.02 (0.54)	2.92 (0.23)	3.09 (0.49)	2.94 (0.46)
*F‐*statistic	1.92			0.23			0.46		
*p*‐value	.16			.80			.64		

Abbreviations: Ic = informal carer; p = older person; Hcn = home care nurse.

* *F‐*statistic significant at .01 level.

## 4 DISCUSSION

This study shows that in triads of older persons, informal carers and home care nurses, perceptions can both align and misalign regarding the care providers’ level of trust in the older person and the person's view of their own behaviour. Notably, most triads had misalignments, with informal carers or nurses having a lower level of trust in the person while the older persons viewed their own behaviour towards either care provider positively. Our study shows a negative relationship between the level of alignment between informal carers and older persons and the level of alignment between informal carers and nurses, implying that the level of alignment in one relationship in the triad could impact the level of alignment in another relationship in the triad. Therefore, our study supports the importance of taking triadic relationships and interactions as the starting point when analysing the interpersonal character of trust instead of examining individuals in isolation (Kenny et al., 2006). For example, Murray and McCrone (2015) show the importance of alignment in the expectations of informal carers or nurses and the behaviour of (older) persons, as alignment may enhance the quality and stability of mutual relationships. The misalignment between informal carers' and nurses' level of trust and the persons' view of their own behaviour could be explained by the fact that trust building depends on the levels of longevity and continuity of care and the extent to which positive interactions occur over time (Kramer & Cook, 2004), such as happens when a regular formal care provider frequently visits the person (Becker & Roblin, 2008; Bova, Fennie, Watrous, Dieckhaus, & Williams, 2006; Cunningham, Sohler, Korin, Gao, & Anastos, 2007). Research by Lindahl and colleagues (2011) indicates that facilitating contact between individuals is important in creating a sense of familiarity that could lead to the establishment of a trusting relationship. In our study, the nurses spent about 20 min with each older person. This limitation, as well as the restricted opportunity for positive interaction between the nurse and older person and possibly also between nurse and informal carer (who does not live with the person) could reduce the time available to build mutual trusting relationships in these triads.

Moreover, the perception of care providers on trusting the person could have been based on the person's (non)verbal cues. Kramer (1996, 2004) suggests that care providers act as ‘intuitive auditors’ when assessing a person's behaviour. Their assessment could be based on two dimensions of trust that are similar to a person's assessment of trust in their care provider (Hall et al., 2001). These two dimensions are as follows: honesty (persons expressing benign motives, acting cooperatively and describing situations without exaggeration or ignoring relevant facts) and competence (assertiveness, compliance with directives and identifying and providing relevant information) (Kramer & Cook, 2004; Pelaccia et al., 2016; Rogers, 2002). Therefore, the care providers in this study could have picked up on non‐verbal cues in their daily interactions with an older person, which could explain their level of trust in that person.

Research into other concepts such as reporting pain and self‐efficacy suggests that (older) person and care provider characteristics could influence the level of alignment. However, contradictory results can be found with regard to which characteristics significantly influence alignment and whether misalignment can be attributed to lower or higher scores for the older person or care provider (Green, Wells, & Laakso, 2011; Hoth et al., 2007; Li & Loke, 2014; McCarthy & Lyons, 2015; Porter et al., 2002; Shega, Hougham, Stocking, Cox‐Hayley, & Sachs, 2004). For example, various studies show that the severity of the person's health condition is related to misalignment between older persons and informal carers, but this could mean that (older) persons or informal carers either overestimate or underestimate the situation (Hoth et al., 2007; Porter et al., 2002). Our study, for example, suggests a worse health condition of older persons in triads with misalignment if informal carers have a higher level of trust than the patient's view of their own behaviour. This suggests that specific older person and care provider characteristics could explain the alignment level. However, given the contradictory findings of other studies, future research is necessary to explore in depth which older person and care provider characteristics influence trust and the relationship between those characteristics and the alignment categories.

### 4.1 Relationship between trust in persons and self‐management

The second aim of this study was to explore the relationship between care providers' trust in older persons and the person's management of their own health. In the triads, the level of trust of the informal carer and the nurse in the older person was unrelated to the person's self‐rated ability to self‐manage, but did relate to their own perceptions of the person's self‐management behaviour. This study shows that informal carers view the person's self‐management ability more positively when their level of trust in the person aligns with the level of trust of the nurse.

Firstly, it is important to mention that the lack of relationship between trust in older persons and the person's self‐rated self‐management behaviour could be explained by the fact that the latter is determined not only by the quality of the relationships between older persons and care providers, but also by person‐specific characteristics such as state of health or financial situation (Barlow et al., 2002; Newman, Steed, & Mulligan, 2004),

Secondly, from a relational point of view, the explanation could lie in a gap between intrinsically *believing* in trusting a person and *expressing* that trust. Communication is crucial for trust building, and being open and honest could help generate trust between individuals (Calnan & Rowe, 2008). Research indicates that the extent to which persons experience trust from their care providers influences the trust they have in their own competence (Kramer & Cook, 2004; Thorne & Robinson, 1988; Wilk & Platt, 2016). Becker and Roblin (2008) showed that relationships with formal primary care providers who the (older) person perceives as honest and supportive are associated with better self‐management, while relationships that are perceived as unsupportive or strained lead to worse self‐management. Trusting, therefore, demonstrates caring for the patient as a person. More importantly, care providers need to tell their care recipients that they believe the person can indeed manage their health. Inspiring an optimistic attitude can boost a person's confidence in their ability to manage their health (Gabay, 2015; Kramer & Cook, 2004).

### 4.2 Study limitations

When interpreting the results, careful consideration must be paid to the following. First, due to the cross‐sectional design, we cannot draw conclusions on the causality between the informal carers' and nurses' levels of trust in the older person's behaviour and the person's view on their own behaviour. Also, we cannot draw conclusions on the causality between the levels of trust in the person and the person's self‐management of their health.

Second, the relatively low number of triads in this study could be a limitation. However, this study aimed to explore the under‐researched topic of care providers’ trust in older persons and was intended to provide initial insight into the concept from three perspectives, as well as the relationship of trust to older persons' self‐management ability.

Third, because we focused on analysing triads, the older persons were nested in with the informal carers and nurses. We are aware that multilevel analysis is commonly used with nested data. Unfortunately due to our small sample size, we were unable to perform such analysis. However, given the difficulty of collecting data from older persons – face‐to‐face interviewing was necessary – we believe our study still provides a good first insight into a relatively new research topic for a relevant study group.

Fourth, careful consideration must be taken in the generalisability of our results. Although older persons were initially sampled at random by the home care organisation, the final study sample could be biased as it depended on the older person's consent to sharing their contact details and participating in the study. As a result, informal carers and nurses were sampled non‐randomly. And because of this, the sample possibly does not represent all types of respondents, ignoring ethnic diversity, which could have affected our results. For example, Suurmond, Rosenmöller, Mesbahi, Lamkaddem, and Essink‐Bot (2016) and Wezel et al. (2016) show that specific ethnic groups (e.g. Turkish persons) often have a strong informal care network and use less home care. Also, EQ‐5D‐3L utility scores for older persons were significantly lower than the Dutch population norms.

Fifth, as 18 nurses filled in different questionnaires, it is possible that these nurses unknowingly compared patients, which could have affected their scoring. Nonetheless, our study specifically focused on analysing triads rather than unconnected groups of individuals in order to analyse the interpersonal character of trust. Therefore, non‐random sampling was necessary.

Sixth, Cronbach's alphas of the adapted older person version of the Physician Trust in the Patient Scale were relatively low, which could be due to the relatively few older persons participating in the study. However, Cronbach's alphas were high for the informal carer and nurse questionnaires, suggesting that the scale is reliable. Seventh, the significant relationships found in the care provider's level of trust and their view of the person's self‐management ability might be slightly overestimated due to common source bias. However, the correlations were relatively low, suggesting that this bias had only a minor effect.

### 4.3 Implications

As both alignment and misalignment occur in triads, this study suggests that building and maintaining trusting relationships between older persons, their informal carer and the nurse is vital. Following relational coordination theory (Gittell, 2002; Weinberg, Lusenhop, Gittell, & Kautz, 2007), the quality of communication between individuals could positively affect the quality of their mutual relationships, and vice versa, and both could be supported by organising informal meetings to discuss mutual expectations regarding the person's behaviour and mutual trust levels (Hartgerink et al., 2014). Such discussions could form part of the regular meetings on an older person's care plan.

It is challenging to build a trusting relationship between a person and a specific nurse or between an informal carer and a nurse because home care is often delivered by a team of nurses (Weman & Fagerberg, 2006; Wiechula et al., 2016). Therefore, we suggest that home care organisations should try to limit the number of nurses delivering care to one person and ideally should strive to pair persons and nurses, although we realise this is not always feasible in practice.

Expressing trust in persons is important and this value should be reflected in the attitudes and actions of informal carers and nurses (Kim et al., 2004; Wiechula et al., 2016). To achieve this, we suggest that nurses could benefit from ‘compassionate assessment’ sessions, in which a group of nurses, guided by a trainer, reflect on building relationships with persons and informal carers (Adam & Taylor, 2014; Wiechula et al., 2016). Compassion is central to how people perceive care through relationships based on empathy, respect and dignity and primarily involves being aware of someone's feelings and interacting with them in a meaningful way (Dewar, Pullin, & Tocheris, 2011). Expressing compassion in a caring relationship could enhance a feeling of being trusted by another person (LoCurto & Berg, 2016) and compassionate training could help nurses to express their compassion towards older persons more explicitly (Adam & Taylor, 2014; Dewar et al., 2011). Although this type of training is usually for student nurses (Adam & Taylor, 2014), it could help more experienced nurses as it would enable them to reflect on their own behaviour (Gould et al., 2018). Nurses who have had this training might also be better able to assess and discuss the informal carer's attitude and behaviour towards the person.

This study took a first step in exploring the level of trust that informal carers and nurses have in older persons. Future research could take the next steps. First, longitudinal studies, observing older person–care provider interactions over time, could produce useful insight into how trust develops in daily practice (LoCurto & Berg, 2016). Second, future studies could look at the trust levels of other care providers (general practitioners, physiotherapists) and social network members (family members and friends), as older persons often receive care from many individuals whose (social) support has an impact on their self‐management ability (Doekhie, Buljac‐Samardzic, Strating, & Paauwe, 2017; Rogers, Vassilev, Brooks, Kennedy, & Blickem, 2016; Vassilev, Rogers, Kennedy, & Koetsenruijter, 2014). Regardless of the type of research, future studies should consider that studying trust may lead to ethical issues between individuals, for example distrust. Although our study did not identify distrust between respondents, Kramer and Cook (2004) show that it can occur between patients and care providers, making it important to take into account when conducting research on trust.

## 5 ETHICAL CONSIDERATIONS

The study protocol (protocol number MEC‐2017‐207) was reviewed by the medical ethics committee of the Erasmus Centre Rotterdam in the Netherlands. The Medical Research Involving Subjects Act was inapplicable, so further examination was waived.

## CONFLICT OF INTEREST

There is no conflict of interest to declare.

## AUTHORS’ CONTRIBUTIONS

All authors (K.D., M.B.S., M.S. and J.P.) designed the study. K.D. collected the data together with V.B. and M.O. All authors frequently discussed the appropriate methods for analysis and results of the analysis. K.D. drafted the first version of the manuscript and based on input and reformulations of sentences of M.B.S., M.S. and J.P., K.D. revised the manuscript. All authors read and approved the definitive version of the manuscript and agree to be accountable for all aspects of the study.
