# Supplementary material for: Trust in older persons: A quantitative analysis of alignment in triads of older persons, informal carers and home care nurses
Source: Health Soc Care Community. 2019 Jul 26;27(6):1490–506. doi: 10.1111/hsc.12820 (PMC6852099; doi:10.1111/hsc.12820)
Supplement: Supplementary file 1 [file HSC-27-1490-s001.docx]

| **Supporting material 1. Descriptive characteristics of the triads per (mis)alignment category of table 3** | | | | | | | | | |
| --- | --- | --- | --- | --- | --- | --- | --- | --- | --- |
| **1. Informal care provider – older person dyad** | | | | | | | | | |
|  | Informal care provider < older person (n=18) | | | Alignment informal care provider – older person (n=18) | | | Informal care provider > older person (n=2) | | |
|  | P | Icp ^a^ | Hcn^b^ | P | Icp | Hcn | P | Icp | Hcn |
| **Age**, mean (SD) | 78.44 (8.79) | 61.78 (13.94) | 42.40 (11.69) | 83.28 (6.31) | 64.12 (18.34) | 46.59 (11.31) | 78.50 (4.95) | 58.00 (4.24) | 37.00 (4.24) |
| **Gender male**, N (%) | 8 (44.40) | 4 (22.20) | 0 | 7 (38.9) | 7 (38.90) | 1 (5.6) | 1 (50.00) | 1 (50.00) | 0 |
| **Educational background,** N (%) |  |  |  |  |  |  |  |  |  |
| Less than secondary school | 6 (33.30) | 4 (22.20) |  | 1 (5.60) | 4 (22.2) |  | 1 (50.00) | 1 (50.00) |  |
| Secondary school / technical school | 10 (55.60) | 12 (66.70) | 16 (94.10) | 17 (94.40) | 11 (61.10) | 16 (88.90) | 1 (50.00) | 1 (50.00) | 2 (100) |
| College or above | 2 (11.10) | 2 (11.10) | 1 (5.3) | 0 | 3 (16.70) | 2 (11.10) | 0 | 0 |  |
| **EQ-5D-3L utility score,** mean (SD) | 0.54 (0.33) |  |  | 0.48 (0.26) |  |  | 0.54 (0.03) |  |  |
| **EQ-5D VAS score**, mean (SD) | 55.00 (11.88) |  |  | 58.89 (14.20) |  |  | 40.00 (0.00) |  |  |
| **Co-resident informal care provider** (yes), N (%) | 7 (38.90) |  |  | 8 (44.44) |  |  | 0 |  |  |
| **Relationship to older person,** N (%) |  |  |  |  |  |  |  |  |  |
| Partner |  | 7 (41.20) |  |  | 7 (38.90) |  |  |  |  |
| Son/daughter (in law) |  | 8 (47.10) |  |  | 9 (50.00) |  |  | 1 (50.0) |  |
| Grandson/granddaughter (in law) |  |  |  |  | 1 (5.60) |  |  |  |  |
| Nephew/niece/cousin |  | 1 (5.90) |  |  | 0 |  |  |  |  |
| Friend |  | 1 (5.90) |  |  | 0 |  |  | 1 (50.0) |  |
| Neighbour |  |  |  |  | 1 (5.60) |  |  |  |  |
| **Average days per week home care,** mean (SD) | 5.03 (2.53) |  |  | 4.10 (2.76) |  |  | 3,15 (1.56) |  |  |
| **Average number of home care nurses per week,** mean (SD) | 6.37 (3.52) |  |  | 4.36 (1.96) |  |  | 3.00 (0) |  |  |
| **Average time (in minutes) per home care nurse visit,** mean (SD) | 19.93 (12.03) |  |  | 16.61 (12.34) |  |  | 5.00 (7.07) |  |  |
|  | | | | | | | | | |
| **2. Home care nurse – older person dyad** | | | | | | | | | |
|  | **Home care nurse < older person (n=23)** | | | **Alignment home care nurse – older person (n=6)** | | | **Home care nurse > older person (n=10)** | | |
|  | **P** | **Icp ^c^** | **Hcn** | **P** | **Icp** | **Hcn** | **P** | **Icp** | **Hcn** |
| **Age**, mean (SD) | 79.78 (8.01) | 62.33 (14.27) | 42.70 (10.78) | 50.17 (13.15) | 50.20 (23.92) | 81.50 (4.04) | 81.10 (10.04) | 69.40 (9.78) | 45.11 (12.39) |
| **Gender male**, N (%) | 6 (26.10) | 8 (36.40) | 1 (4.30) | 3 (50.00) | 1 (16.70) | 0 | 7 (70.00) | 3 (30.00) | 0 |
| **Educational background,** N (%) |  |  |  |  |  |  |  |  |  |
| Less than secondary school | 6 (26.10) | 7 (31.80) | 19 (86.40) | 0 | 0 | 0 | 2 (20.00) | 2 (20.00) | 0 |
| Secondary school / technical school | 16 (69.60) | 13 (59.10) | 3 (13.60) | 6 (100) | 6 (100) | 6 (100) | 7 (70.00) | 5 (50.00) | 10 (100) |
| College or above | 1 (4.30) | 2 (9.10) | 0 | 0 | 0 | 0 | 1 (10.00) | 3 (30.00) | 0 |
| **EQ-5D-3L utility score,** mean (SD) | 0.48 (0.31) |  |  | 0.69 (0.20) |  |  | 0.45 (0.27) |  |  |
| **EQ-5D VAS score**, mean (SD) | 53.70 (12.63) |  |  | 63.33 (15.38) |  |  | 55.50 (13.43) |  |  |
| **Co-resident informal care provider** (yes), N (%) | 6 (26.09) |  |  | 2 (33.33) |  |  |  | 7 (70.00) |  |
| **Relationship to older person,** N (%) |  |  |  |  |  |  |  |  |  |
| Partner |  | 6 (26.10) |  |  | 2 (33.33) |  |  | 6 (60.00) |  |
| Son/daughter (in law) |  | 12 (52.20) |  |  | 3 (50.00) |  |  | 3 (30.00) |  |
| **G**randson/ granddaughter (in law) |  |  |  |  | 1 (16.78) |  |  |  |  |
| Nephew/niece/cousin |  | 1 (4.30) |  |  |  |  |  |  |  |
| Friend |  | 2 (8.70) |  |  |  |  |  |  |  |
| Neighbour |  |  |  |  |  |  |  | 1 (10.00) |  |
| **Average days per week home care,** mean (SD) | 5.28 (2.25) |  |  | 4.17 (3.25) |  |  | 3.40 (2.95) |  |  |
| **Average number of home care nurses per week,** mean (SD) | 5.59 (3.39) |  |  | 5.00 (2.00) |  |  | 5.00 (3.16) |  |  |
| **Average time (in minutes) per home care nurse visit,** mean (SD) | 20.44 (10.33) |  |  | 9.67 (7.12) |  |  | 22.50 (18.75) |  |  |
|  |  |  |  |  |  |  |  |  |  |
| **3. Home care nurse – informal care provider dyad** | | | | | | | | | |
|  | **Home care nurse < informal care provider (n=5)** | | | **Alignment home care nurse – informal care provider (n=14)** | | | **Home care nurse > informal care provider (n=19)** | | |
|  | **P** | **Icp** | **Hcn** | **P** | **Icp** | **Hcn** | **P** | **Icp ^d^** | **Hcn ^d^** |
| **Age**, mean (SD) | 81.40 (4.93) | 54.60 (5.68) | 47.40 (10.33) | 82.14 (6.76) | 65.92 (20.21) | 41.00 (10.24) | 79.53 (9.09) | 62.44 (13.20) | 46.07 (12.50) |
| **Gender male**, N (%) | 3 (60.00) | 1 (20.00) | 0 | 2 (14.30) | 7 (50.00) | 1 (7.10) | 11 (57.90) | 4 (21.10) | 0 |
| **Educational background,** N (%) |  |  |  |  |  |  |  |  |  |
| Less than secondary school | 1 (20.00) | 2 (40.00) | 0 | 2 (14.30) | 3 (21.43) | 0 | 4 (21.10) | 0 | 0 |
| Secondary school / technical school | 4 (80.00) | 3 (60.00) | 5 (100) | 12 (85.70) | 10 (71.43) | 12 (85.70) | 11 (57.90) | 17 (94.40) | 17 (94.40)) |
| College or above | 0 | 0 | 0 | 0 | 1 (7,14) | 2 (14.30) |  | 1 (5.60) | 1 (5.60 |
| **EQ-5D-3L utility score,** mean (SD) | 0.59 (0.20) |  |  | 0.38 (0.33) |  |  | 0.60 (0.25) |  |  |
| **EQ-5D VAS score**, mean (SD) | 54.00 (16.73) |  |  | 55.36 (12.16) |  |  | 57.11 (13.78) |  |  |
| **Co-resident informal care provider** (yes), N (%) | 0 |  |  | 6 (42.86) |  |  | 9 (47.37) |  |  |
| **Relationship to older person,** N (%) |  |  |  |  |  |  |  |  |  |
| Partner |  | 0 |  |  | 6 (46.86) |  |  | 8 (42.11) |  |
| Son/daughter (in law) |  | 4 (80.00) |  |  | 6 (46.86) |  |  | 8 (42.11) |  |
| **G**randson/ granddaughter (in law) |  |  |  |  | 1 (7.14) |  |  |  |  |
| Nephew/niece/cousin |  |  |  |  |  |  |  | 1 (5.26) |  |
| Friend |  | 1 (20.00) |  |  |  |  |  | 1 (5.26) |  |
| Neighbour |  |  |  |  |  |  |  | 1 (5.26) |  |
| **Average days per week home care,** mean (SD) | 3.50 (2.11) |  |  | 5.21 (2.45) |  |  | 4.42 (2.79) |  |  |
| **Average number of home care nurses per week,** mean (SD) | 4.25 (1.89) |  |  | 5.00 (2.35) |  |  | 6.13 (3.85) |  |  |
| **Average time (in minutes) per home care nurse visit,** mean (SD) | 10.00 (6.12) |  |  | 20.71 (11.07) |  |  | 20.16 (15.13) |  |  |
| ^a^ N= 17 for the informal care providers’ relationship to patient due to missing data on this variable for one informal care provider  ^b^ N = 17 for the home care nurses’ educational level due to missing data on this variable for one nurse  ^c^ N = 22 for the informal care providers’ educational level due to one missing data and n= 21 for the relationship to older person due to missing data for two informal care providers  ^d^ N = 18 for the informal care providers’ and nurses’ educational level due to missing data for one in both groups | | | | | | | | | |
